# Epidémiologie et prise en charge des infections du per-partum à la maternité du centre hospitalier départemental de l'Ouémé-Plateau au Bénin

**DOI:** 10.11604/pamj.2014.17.89.2857

**Published:** 2014-02-04

**Authors:** Jacques Saizonou, Laurent Ouédraogo, Moussiliou Noël Paraiso, Paul Ayélo, Alphonse Kpozèhouen, René Daraté, Esther Traoré

**Affiliations:** 1 l'Institut Régional de Santé Publique de Ouidah/ Bénin; 2 Faculté des sciences de la Santé de l'Université d'Abomey-Calavi du Bénin; 3 Ministère de la santé du Bénin; 4 Bureau pays de l'OMS au Bénin

**Keywords:** Infection du per-partum, incidence, prise en charge, Bénin, intrapartum Infection, incidence, management, Bénin

## Abstract

**Introduction:**

La présente étude avait pour objectif de mesurer l'ampleur des infections du per-partum à la maternité du Centre Hospitalier Départemental de l'Ouémé-Plateau d'analyser les conditions de survenue et de prise en charge dans la perspective de l'amélioration des soins et services maternels et néonataux.

**Méthodes:**

Une étude descriptive et analytique a été conduite de Août 2009 à Février 2010 et a impliqué 110 mères victimes d'infections du per-partum; elles ont été soumises à un questionnaire durant tout leur séjour hospitalier; des observations des pratiques de prise en charge des prestataires de soins ont été menées et des registres et autres supports de données sanitaires ont été exploités. L'analyse des données a été effectuée à l'aide du logiciel Epi Info 6.4 dans sa version française.

**Résultats:**

L'incidence des infections du per-partum était de 5,9 pour 100 accouchements (110/ 1875); les types d'infections les plus incriminés étaient les endométrites (27,3%), les chorioamniotites (18,2%), les infections du site opératoire (12,7%) et les infections urinaires (2,7%). Les examens cliniques ont été relativement acceptables. En revanche, les examens biologiques ont été très insuffisants expliquant l'imprécision importante au niveau des diagnostics. L'antibiothérapie a été instaurée systématiquement. Le taux de mortinaissance était de 25,6% et les enfants de petit poids représentaient 23,2%.

**Conclusion:**

L'incidence des infections du per-partum dans la présente étude était assez élevée. Des efforts de préventions et de dépistage de ces infections sont nécessaire afin d'améliorer la qualité des soins

## Introduction

Les infections occupent le deuxième rang (15%) parmi les causes contributives de décès maternels dans le monde [1]. Les infections graves comptent pour 36% de tous les décès de nouveau-né et 86% des décès néonataux résultent directement de trois grandes causes: - les infections graves, l'asphyxie et la prématurité. Elles représentent la principale cause de mortalité néonatale après la première semaine de vie [2]. Les infections sont prépondérantes surtout dans les pays en développement où les soins et l'environnement des soins sont de qualité insuffisante. Les infections pendant la grossesse et l'accouchement ont longtemps été redoutables et à la base des secteurs d'isolement dans les maternités en particulier contre l'infection puerpérale. L'agent infectieux peut être un virus (rubéole, hépatite virale, herpès, varicelle, VIH/ SIDA), une bactérie (streptocoque B, gonococcie, rickettsioses), un parasite (toxoplasmose) ou un autre micro-organisme. L’étiologie de l'endométrite, une des complications après l'accouchement, est polymicrobienne [3]. Les infections sont souvent la conséquence d'une mauvaise hygiène au cours de l'accouchement ou de maladies sexuellement transmissibles non traitées. Les soins dispensés systématiquement pendant le per-partum permettent de dépister rapidement ces infections et de les traiter par l'administration d'antibiotiques. Le risque d'infection est également élevé à l'hôpital, à cause du nombre important de gestes favorisant l'entrée de bactéries (examens vaginaux, sondage urinaire, césarienne). L'effort principal de la prévention est d'assurer un accouchement dans de bonnes conditions d'hygiène, comme il est conseillé dans le package Mère-enfant [4]. La présente recherche a pour but de mesurer l'ampleur des infections du per-partum à la maternité du Centre Hospitalier Départemental de l'Ouémé-Plateau (CHD-OP) et d'analyser les conditions de survenue et de prise en charge dans la perspective de l'amélioration des soins et services maternels et néonatals.

## Méthodes

**Cadre d’étude:** le Centre hospitalier départemental de l'Ouémé-Plateau (CHD-OP), est une structure sanitaire publique d'une capacité de 355 lits, située dans la ville de Porto-Novo, capitale du Bénin. La maternité d’étude est un des services de cet hôpital qui dessert une population estimée à 1.475.500 habitants en 2010. Elle est dotée de 123 lits et sert de référence à sept maternités chirurgicales et 67 maternités périphériques. Les femmes en âge de procréer dans la zone couverte étaient au nombre de 372.600 et le nombre de grossesses attendues est de 66.700 selon les données du système national d'information sanitaire de 2010 [5]. Le personnel de soins est composé de six gynécologues obstétriciens, 28 sages-femmes, 17 infirmiers/ infirmières, 22 aides soignants. La maternité du CHD-OP mène les activités de soins et service, de formation et de recherche.

**Type d’étude:** Il s'agit d'une étude transversale descriptive et analytique qui s'est déroulée au CHD-OP d'Août 2009 à Février 2010.

**Populations cibles:** Cette étude a concernée 110 mères admises pour les infections du per partum et le personnel de soins: sages femmes, gynéco-obstétriciens, infirmiers (ères) chargés des soins et services.

**Méthode et technique d’échantillonnage:** Toute femme qui a développé une infection du per-partum et qui a accepté par consentement éclairé de participer à l’étude a été retenue. L'infection du per-partum a été définie comme toute fièvre survenant chez toute femme au cours du per-partum à l'exclusion de toute fièvre d'origine palustre. Le per-partum a été défini comme la période allant de six mois de grossesse à sept jours après l'accouchement.

**Variables de l’étude:** la variable dépendante a été l'incidence de l'infection du per-partum. Les variables indépendantes ont été: les caractéristiques sociodémographiques; les types et moments de survenue de l'infection; les éléments de prise en charge et l'issue des accouchements.

**Techniques et outils de collecte:** un questionnaire a été administré aux mères admises et traitées pour infections du per-partum pour recueillir les données concernant leurs caractéristiques sociodémographiques, l’état à l'admission, les antécédents, les éléments de leur prise en charge. Les registres d'admission, d'accouchement, d'hospitalisation, du bloc opératoire et les dossiers médicaux des patientes ont été exploités; le seul questionnaire a été rempli depuis l'admission de la mère jusqu’à sa sortie, sept jours après l'accouchement. Des entretiens individuels avec les prestataires de soins ont été réalisés pour compléter les données manquantes sur les supports de données disponibles. Des observations à l'aide d'un guide d'observation, ont été effectuées pour apprécier les pratiques des personnels de soins en matière d'actes techniques de prise en charge de ces infections du per-partum. La collecte des données a été assurée par une équipe composée d'un gynéco-obstétricien, de deux sages-femmes sous la supervision de l'auteur principal de ce travail.

**Analyse des données:** les outils de collecte ont été codifiés après vérification de la complétude des données. Une double saisie des données quantitatives a été effectuée avec le logiciel Epi info 6.4. Les données qualitatives ont été saisies à l'aide du logiciel Word. Les données quantitatives ont été exprimées en proportions; des croisements de la variable dépendante (présence d'infection du per-partum) avec les variables explicatives ont été effectués pour apprécier les relations entre elles. Le test Khi deux de Pearson a été appliqué au seuil de signification fixé à 0,05.

## Résultats

### Caractéristiques sociodémographiques et obstétricales des mères ayant développé une infection du per-partum (Tableau 1, Tableau 2)

Un total de 110 mères qui ont développé une infection du per-partum, a été recensé pendant la période d’étude sur les 1875 femmes admises pour accouchement pendant la période d’étude, soit une incidence de l'infection du per-partum de 5,9%. La plupart des mères (80,9%), était de la tranche d’âge de 19 à 34 ans. Les revendeuses de divers produits ont été les plus représentées (48,2%) suivies des artisanes (15,5%). La moitié des mères (50,9%) était non scolarisée, suivies de celles ayant le niveau primaire (31,8%). La moitié des mères (50,8%), pratiquait le christianisme, suivie de celles de la religion islamique (18,2%). Les mères mariées étaient majoritaires, 87,2% versus 12,8% pour les non mariées; les mères en provenance du milieu urbain étaient les plus représentées 62,7% versus 37,3% pour celles du milieu rural.

**Tableau 1:** Caractéristiques sociodémographiques des mères ayant développé une infection du per-partum

Variables	N = 110	%
**Age**		
Moins de 18 ans	8	7,3
19 – 34 ans	89	80,9
35 ans & +	13	11,8
**Profession**		
Revendeuse	53	48,2
Artisane	17	15,5
Femmes au foyer	16	14,5
Autres	24	18,8
**Niveau instruction**		
Non scolarisée	56	50,9
Primaire	35	31,8
Secondaire	19	17,3
**Religion**		
Christianisme	56	50,8
Islamique	20	18,2
Autres	34	31,0
**Situation matrimoniale**		
Mariée	96	87,2
Autres	14	12,8

**Tableau 2:** Caractéristiques sociodémographiques des mères ayant développé une infection du per-partum (suite)

Variables	N = 110	%
**Résidence**		
Rural	41	37,3
Urbain	69	62,7
**Parité**		
0 - 2 enfants	80	72,7
3 - 4 enfants	21	19,1
5 enfants et +	9	8,2
**Nombre d'enfants vivants**		
0 - 2 enfants	83	75,5
3 - 4 enfants	24	21,8
5 enfants et +	3	2,7
**Issues des dernières grossesses**		
Accouchement voie basse	96	87,3
Césarienne	14	12,7
**Issues dernières naissances**		
Né vivant	106	96,4
Mort-né	4	3,6

Les mères ayant accouché moins de deux enfants étaient majoritaires, 75,5% suivies de celles ayant 3-4 enfants, 21,8%. Les accouchements s’étaient terminés pour la plupart par voie basse, 87,3%, et des césariennes, 12,7%. Ces accouchements s’étaient terminés par des mort-nés, 3,6% versus 96,4% de naissances vivantes.

### Etat et conditions d'admission des mères à la maternité du CHD-OP (Tableau 3)

Les mères ayant développé une infection du per-partum étaient majoritairement conscientes à l'admission (94,4%). Elles étaient pour plupart, 80,0%, évacuées à partir d'autres formations sanitaires. Parmi ces évacuées, 62 (70,5%) venaient des centres de santé publics contre 26 (29,5%) qui venaient des cabinets et cliniques privés. Les raisons de la référence étaient la température élevée (70,5%), et les antécédents de césarienne (17,0%). La rupture prématurée des membranes avant l'admission a été observée chez 36 mères (32,7%) dont le délai entre la rupture et l'accouchement était plus de 48 heures chez 26 mères (72,2%) versus moins de 48 heures chez 10 mères (27,8%).

**Tableau 3:** Etat et conditions d'admission des mères développant des infections du per-partum

Variables	N = 110	%
**Etat à l'admission**		
Conscient	106	96,4
Inconscient	4	3,6
**Mode d'entrée**		
Evacuée	88	80,0
Venue du domicile	22	20,0
**Rupture prématurée des membranes**		
Non	74	67,3
Oui	36	32,7

### Types d'infections et issues de la grossesse (Tableau 4)

Le type d'infection dominant présenté par les mères était l'endométrite (27,3%). L'infection était survenue le plus souvent pendant le travail d'accouchement (40,9%). La grossesse chez ces mères s’était terminée par un accouchement à terme (84,5%). Les mortinaissances issues des accouchements représentaient 25,5% parmi lesquelles les mort-nés macérés représentaient 53,6%. Les enfants de petit poids chez les naissances vivantes représentaient 23,2% (19/ 82).

**Tableau 4:** Types d'infections du per-partum et issues de la grossesse

Variables	110	%
**Type d'infection du per-partum**		
Endométrite	30	27,3
Chorioamniotite	20	18,2
Infection du site opératoire	14	12,7
Infection urinaire	3	2,7
Pyélonéphrite	2	1,8
Infections non précisées	41	37,3
**Moment de survenue de l'infection du per-partum**		
Pendant le travail	45	40,9
A l'entrée	34	30,9
En hospitalisation	31	28,2
**Issue grossesse actuelle**		
Accouchement à terme	93	84,5
Accouchement prématuré	7	6,4
Autres	10	9,1
Etat enfant	N	%
Né vivant	82	74,5
Mort-né	28	25,5

### Prise en charge des mères ayant présenté des infections du per partum (Tableau 5, Tableau 6)

Gestes spécifiques d'urgence réalisés: la prise de constances la plus réalisée a été la mesure de la TA (92,7%). Le touchez vaginal et la pose de la sonde à demeure ont été les gestes cliniques les plus exécutés, 54,5%. La rupture artificielle de la poche des eaux a été effectuée chez 28,2% des mères, autant chez les mères du milieu urbain que du milieu rural.

**Tableau 5:** Eléments de prise en charge des mères

Variables	Urbain	Rural	Total N = 110	%	P value
	n = 69 %	n = 41 %			
**Gestes spécifiques**					
Mesure de la T.A.	94,2	90,2	102	92,7	0,69
Mesure du pouls	84,1	78,0	90	81,8	0,62
Mesure la température	91,3	85,4	98	89,1	0,49
Fréquence respiratoire	75,4	73,2	82	74,5	0,98
Toucher vaginal	56,5	51,2	60	54,5	-
Examen sous valve	2,9	2,4	3	2,7	-
Ponction Douglas	21,7	22,0	24	21,8	-
Sonde à demeure	56,5	51,2	60	54,5	-
Rupture artificielle de la poche des eaux	30,4	24,4	31	28,2	-
Prélèvement vaginal	21,7	12,2	20	18,2	-
**Examens de sang**					-
Groupage sanguin/Rhésus	89,9	90,2	99	90,0	0,43
NFS	89,9	87,8	98	89,1	0,43
TPHA	1,4	0,0	1	0,9	-
VDRL	2,9	0,0	2	1,8	-
Bactéries	0,0	0,0	0	0,0	-
Goutte épaisse et DP	0,0	7,3	3	2,7	-

**Tableau 6:** Eléments de prise en charge des mères (suite)

Variables	Urbain	Rural	Total N = 110	%	P value
	n = 69%	n = 41%			
**Examens d'urines**					
ECBU	4,3	0,0	3	2,7	-
Leucocytes	0,0	2,4	1	0,9	-
Bactéries	0,0	4,9	2	1,8	-
**Soins médicaux réalisés**					-
Antibiotiques	92,8	90,2	101	91,8	0,63
Transfusion sanguine	10,1	12,2	12	10,9	-
Antipyrétiques	47,8	36,6	48	43,6	-
Curage	4,3	0,0	3	2,7	-
Accouchement voie basse	23,2	22,0	25	22,7	-
**Interventions obstétricales**					-
Césarienne	73,9	68,3	79	71,8	0,72
Révision utérine sous AG	5,8	9,8	8	7,3	-
Laparotomie pour RU	0,0	2,4	1	0,9	-
Hystérectomie	0,0	2,4	1	0,9	-
Curetage	1,4	4,9	3	2,7	-

**Examens de sang réalisés:** le groupage sanguin/ Rhésus et la Numération Formule Sanguine (NFS) ont été les examens les plus réalisés chez ces mères, respectivement 90,0% et 89,1%; la même tendance a été observée aussi bien chez les mères du milieu urbain que du milieu rural. Les examens les moins réalisés (moins de 2%) ont été les tests de Treponema Pallidum Hemagglutinations Assay (TPHA), de Venereal Disease Research Laboratory (VDRL) et l'hémoculture.

**Examens d'urine réalisés:** les examens d'urines ont été rarement demandés; il s'agit de l'Examen Cytobactériologique des Urines (ECBU), l'antibiogramme, la présence des leucocytes et les bactéries dans les urines

### Soins médicaux et obstétricaux réalisés

La majorité des mères ont bénéficié d'une antibiothérapie, 91,8%, autant pour celles du milieu urbain que du milieu rural. Elles étaient 79 mères (71,8%) à avoir subi la césarienne réalisée autant chez les mères du milieu urbain que celles du milieu rural.

## Discussion

La présente étude conduite à la maternité du CHD de l'Ouémé-Plateau a été menée sur une période de six mois (Août 2009 - Février 2010). Cette période a été jugée appropriée pour apprécier l'importance de cette affection obstétricale ainsi que les éléments de sa prise en charge. En effet, les données sanitaires montrent que le nombre d'accouchements au cours du deuxième semestre de l'année est le plus élevé; dans cette maternité 1875 accouchements ont été enregistrés pour 110 infections du per-partum; ce nombre a été jugé assez suffisant pour les analyses nécessaires. La technique « d'observation participative» a été utilisée pour réduire les biais d'observation liés à la présence d'un enquêteur classique pour la collecte des données. La définition standardisée des concepts relatifs à l'infection du per-partum, la période du per-partum et la fièvre, a permis de réduire les biais liés au recensement des cas.

### Des caractéristiques des mères ayant développé l'infection du per-partum et de leurs conditions d'admission à la maternité

La plupart de ces mères était âgée 19 à 34 ans; ce sont celles de la tranche d’âge qui sont les plus exposées aux activités de maternité, mariées en grande majorité et donc ayant une charge de travail ménagère élevée. Elles étaient pour leur majorité non scolarisées ou de niveau primaire, ayant donc des connaissances et des pratiques limitées et peu favorables à l'application des règles d'hygiène de base. En revanche, la plupart d'entre elles provenait du milieu urbain où les conditions de vie et sanitaires sont jugées meilleures. Les mères ayant moins de deux enfants étaient plus dominantes, ayant donc moins d'expérience en matière d'activité de maternité. La grande majorité de ces mères ont été évacuées à partir des maternités périphériques, sans doute dans des conditions d'hygiène et de transport peu acceptables, sans oublier le délai relativement long pour accéder au centre de référence. Les raisons principales de leur évacuation étaient la température très élevée, signe évocatrice de l'infection.

### De l'incidence des infections du per partum

L'incidence trouvée pour les infections du per-partum était de 5,9 pour 100 accouchements 110/1875). Les types d'infections développés étaient les endométrites (27,3%), les chorioamnionites (18,2%), les infections du site opératoire (12,7%) et les infections urinaires (2,7%). Ce taux d'incidence est relativement élevé, mais se trouve dans les limites de celui rapporté dans la littérature; Sales, dans une étude multicentrique en 2001 organisée par le Comité de Lutte contre les Infections Nosocomiales du Sud-est en France a révélé que 3% des femmes, ayant accouché par voie basse, présentaient une infection, alors que chez les femmes césarisées le taux atteignait 9,3% [6]. Malavaud et al, dans une étude basée sur un recueil systématique et standardisé de données relatives aux nouveaux cas d'infections parmi les femmes accouchées sur une période de trois mois en 2003, ont trouvé un taux de 1,9% (12/615) pour les accouchements par voie basse et de 5,8% (11/189) pour les accouchements par césarienne [7]. Il est à noter que plus des trois quarts des femmes de notre étude ont subi l'opération de la césarienne qui constitue un facteur de risque de survenue de ces infections.

L'endométrite (27,3%) a été le type d'infection le plus incriminé. Dans l’étude multicentrique de Sales, un taux d'incidence supérieur d'endométrite de 50% a été trouvé et elle était 3 à 4 fois plus fréquente après la césarienne qúaprès l'accouchement par voie basse [8–10]. La rupture prolongée de la poche des eaux, un travail d'accouchement de plus de 12 heures, de multiples examens vaginaux sont reconnus comme des facteurs pouvant favoriser la survenue d'une endométrite [11–12]. Dans la présente étude, le tiers des mères a développé une rupture prématurée des membranes et pour les trois quarts d'entre elles, la durée de la rupture excédait 48 heures. L'endométrite pendant la période d'accouchement a été la plus fréquente; ceci s'explique par la présence de plusieurs facteurs de risques pendant cette phase: la rupture prématurée de la poche des eaux, l'environnement de soins, les pratiques des prestataires de soins notamment les multiples examens vaginaux. La chorio-amniotite (18,2%) a été le deuxième type d'infection important développé par les mères. Elle est une infection reconnue fréquente, particulièrement préoccupante par le risque de contamination du liquide amniotique et du foetus. Un grand nombre (7 à 8) de touchers vaginaux favorise la survenue d'une chorio-amniotite chez une femme avec une rupture prématurée des membranes [13]. Ce facteur de risque a concerné près du tiers de nos mères. Il est recommandé que les examens ne doivent pas être inutilement répétés chez ces mères à risque. L'infection du site opératoire (12,7%) a été le troisième type d'infection du per-partum dans notre étude. La fréquence trouvée est largement supérieure à 1,5% après la césarienne dans l’étude de Lemarie et al [14]; mais, elle se trouvait dans la limite supérieure de celui de l'enquête multicentrique de Sales [6], qui avait trouvé une fréquence variant de 1,6 à 18% pour les femmes césarisées. Une étude réalisée au Bénin sur les infections du site opératoire en 2006, a trouvé une fréquence similaire de 13,0% au Centre hospitalier départemental du Zou-Colline [15]. Une revue systématique des infections liées aux soins en Afrique, avait trouvé une fréquence de l'infection du site opératoire variant de 2,5% à 30,9% [16]. L'infection urinaire (2,7%) a été le quatrième type d'infection du per-partum trouvé dans la présente étude. Cette fréquence est proche de 3 à 4% trouvée dans l’étude multicentrique de Sales, et de 5% dans celle de Ovalle et al [17]. L'infection urinaire, favorisée par des facteurs mécaniques et hormonaux, est fréquente chez la femme enceinte.

### De la gestion des infections du per-partum à la maternité du CHD-OP

La majorité des mères ayant développé les infections du per-partum ont bénéficié des examens cliniques de base: mesures de la température, de la tension artérielle, du pouls et de la fréquence respiratoire. Les mères du milieu rural en ont bénéficié autant que leurs homologues du milieu urbain. Quant aux examens obstétricaux, un peu plus de la moitié d'entre elles a subi le toucher vaginal et la pose d'une sonde à demeure; et moins du tiers la ponction de Douglas, la rupture artificielle de la poche des eaux. Si tous ces examens obstétricaux sont utiles pour la prise en charge de ces infections, leur utilisation devra être effectuée le plus rationnellement possible, en prenant soins de ne pas les répéter inutilement, surtout pour les deux premiers gestes (le toucher vaginal et la pose de la sonde à demeure) et en les réalisant dans les conditions d'asepsie optimales possibles [12]; l’évaluation de ces pratiques des professionnels de soins sera développée dans un autre travail à venir. Le prélèvement vaginal a été effectué seulement chez une minorité de ces patientes et autant chez celles du milieu urbain que rural. Pour les examens du sang, très peu en ont bénéficié notamment la recherche de bactéries, les tests du TPHA et du VDRL. Quant aux examens d'urine, la recherche a été aussi pauvre pour ce qui concerne l’étude cytobactériologique de l'urine (ECBU), l'antibiogramme, les bactéries et les leucocytes. Ces résultats montrent que les mesures de dépistage des infections du per-partum ont été très insuffisantes. Cette situation explique le manque de précision observé chez 37,3% (41/110) des mères ayant développé des infections sans un diagnostic précis. Il serait évidemment irrationnel scientifiquement de rendre systématiques tous les examens évoqués ci-dessus et voire d'autres, pour la prise en charge des infections du per-partum. Toutefois, chaque formation sanitaire dans tous pays devra adopter des procédures de prise en charge adéquate de ces infections, sur la base des données scientifiques du moment, compte tenu de leur risque fatal potentiel qu'elles peuvent induire vis-à-vis de la mère et de l'enfant. Des procédures de bonnes pratiques et des recommandations de prévention ont aujourd'hui été écrites, validées, diffusées, comme ce fut le cas des protocoles des services de santé familiale au Bénin [18]. Leur mise en application n'est cependant pas sans poser des difficultés. Les mesures de dépistage de certaines infections sont capitales pour une prévention adéquate des risques. Par exemple, face à la gravité des infections à Streptocoques du groupe B (SGB), il est essentiel de mettre en place une méthode de dépistage à la fois sensible, rapide et simple à utiliser, permettant d'obtenir les résultats pendant l'accouchement, sachant que ces infections sont responsables de 50% d'endométrites [19]. Dans notre étude, les mères ont bénéficié des antibiotiques à titre prophylactique ou curatif; il est admis que l'antibioprophylaxie pendant le travail réduit le risque d'infection materno-foetale surtout à SGB chez les femmes à risque. Une antibioprophylaxie de l'infection est recommandée chez les femmes « à risque » [20]. L'efficacité de l'antibioprophylaxie du per-partum guidée par le résultat du dépistage réduit de 70% le risque infectieux. Elle est proposée en cas de rupture prolongée de la poche des eaux et/ou de la détection de SGB dans la filière génitale maternelle. L'administration d'un antibiotique, seul ou en association permet une réduction significative du nombre de chorioamniotites et d'endométrites [21, 22]. Néanmoins, l'impact de cette antibioprophylaxie large des patientes à « risque » sur l'émergence de germes résistants reste à préciser [24]. Le dépistage systématique des femmes enceintes vis-à-vis du SGB, ainsi que l'antibiothérapie en per-partum sont les mesures les plus efficaces contre les infections néonatales précoces [19]; tel n’était pas le cas dans la présente étude. La revue systématique basée sur les résultats d'un grand nombre d'essais comparatifs randomisés axés sur «l'antibioprophylaxie en cas de césarienne» a conclu à un effet protecteur et des bénéfices importants [25]. L'antibioprophylaxie avant l'accouchement, chez une patiente fiévreuse avec une bactériémie positive au Streptocoque A, a eu des effets bénéfiques pendant le post-partum [26]. Pour la prévention des infections, un des facteurs les plus importants est le temps écoulé entre l'administration des antibiotiques et la survenue de l'infection ou l'initiation de l'intervention chirurgicale comme la césarienne; la réalisation d'une étude serait nécessaire pour apprécier ce facteur. Le taux de mortinaissance était dans la présente étude dix fois plus élevé que celui rapporté par l'ensemble des formations sanitaires du Bénin en 2011 qui est de 2,5% [5] et de 3,4% dans une étude réalisée au Bangladesh en 2008 [27]. Le taux des enfants de petit poids de naissances était également deux fois plus élevé que celui enregistré dans la population générale selon l'enquête démographique et santé [28] et une étude réalisée au Burkina Faso en 2007 [29]. Ces résultats montrent le poids contributif des infections de la mère sur la mortalité f'tale et le développement pondéral des nouveau-nés. Il serait utile de mener des études sur les facteurs associés à la survenue de ces mortinaissances et petits poids chez les mères développant les infections dans les maternités.

## Conclusion

L'infection chez les mères reste une préoccupation majeure au Bénin, essentiellement en raison de la gravité potentielle de l'atteinte maternelle et foetale. La présente étude réalisée dans une maternité chirurgicale au Bénin a montré un taux d'incidence assez élevé des infections du per-partum qui ont eu des effets délétères sur le foetus. L'accès de ces patientes à risque aux antibiotiques a été efectif, mais les mesures de dépistage de ces infections ont été insuffisantes pour la détection des types de germes en cause. Les mesures de prévention et de dépistage sont d'une grande efficacité et constituent souvent une priorité dans la stratégie médicale de lutte contre le risque infectieux chez la femme enceinte. La réalisation d'enquête d'incidence ou de prévalence régulière est indispensable pour évaluer les effets des actions d'information, de sensibilisation et de formation mises en place pour lutter contre les infections développées à l'hôpital.
